# A review on invasions by parasites with complex life cycles: the European strain of *Echinococcus multilocularis* in North America as a model

**DOI:** 10.1017/S0031182021001426

**Published:** 2021-11

**Authors:** Maria A. Santa, Marco Musiani, Kathreen E. Ruckstuhl, Alessandro Massolo

**Affiliations:** 1 Department of Biology, University of Calgary, Alberta T2N 1N4, Canada; 2 Department of Ecosystem and Public Health, Faculty of Veterinary Medicine, University of Calgary, Alberta T2N 4Z6, Canada; 3 Ethology Unit, Department of Biology, University of Pisa, Pisa, 56126, Italy; 4 UMR CNRS 6249 Chrono-Environnement, Université Bourgogne Franche-Comté, Besançon, 25030, France

**Keywords:** Allochthonous strain introductions, biological invasions, complex life cycle, *Echinococcus multilocularis*, emerging infectious diseases, host–parasite interactions, predator–prey interactions

## Abstract

In a fast-changing and globalized world, parasites are moved across continents at an increasing pace. Co-invasion of parasites and their hosts is leading to the emergence of infectious diseases at a global scale, underlining the need for integration of biological invasions and disease ecology research. In this review, the ecological and evolutionary factors influencing the invasion process of parasites with complex life cycles were analysed, using the invasion of the European strain of *Echinococcus multilocularis* in North America as a model. The aim was to propose an ecological framework for investigating the invasion of parasites that are trophically transmitted through predator–prey interactions, showing how despite the complexity of the cycles and the interactions among multiple hosts, such parasites can overcome multiple barriers and become invasive. Identifying the key ecological processes affecting the success of parasite invasions is an important step for risk assessment and development of management strategies, particularly for parasites with the potential to infect people (i.e. zoonotic).

## Graphical abstract

**Figure:**
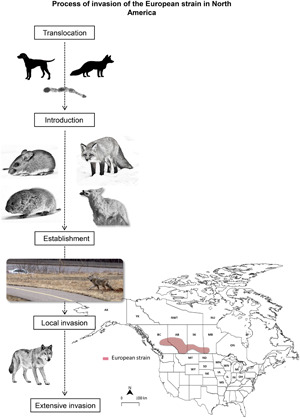

Biological invasions have significant impacts on biodiversity, community structure, and ecosystem processes, often leading to the emergence of diseases that affect animal and human health (Dunn, 2009; Hatcher *et al*., 2012*b*). Changes in species distribution are often associated with human activity and its effects on the environment (Altizer *et al*., 2013; Bellard *et al*., 2016). Therefore, the invasion of parasites is often human-mediated, and co-introductions with their original hosts often give the parasites the chance to exploit new host communities (Prenter *et al*., 2004; Dunn *et al*., 2012). During the invasion process, multiple outcomes can occur: the introduced host species may acquire parasites from their new environment and spill back to native species (Kelly *et al*., 2009); or they can introduce novel parasites into new areas, creating opportunities for the emergence of diseases in native host species (Strauss *et al*., 2012).

Many aetiological agents of emerging infectious diseases (EIDs) (see Glossary in Supplementary Material S1) are considered a significant subgroup of biological invaders due to their rapid increase in incidence and geographic range (Hatcher *et al*., 2012*b*; Ogden *et al*., 2019). In the context of invasion, EIDs primarily arise when a parasite (macro or microparasite) spreads into a new geographical area and host population, or jumps into new host species without prior co-evolutionary history (Dunn and Hatcher, 2015). This can then lead to a disease outbreak or the establishment of new endemism, with enormous conservation, economic, and public health implications (Jones *et al*., 2008; Cunningham *et al*., 2012).

The tapeworm *Echinococcus multilocularis* is the causative agent of human alveolar echinococcosis (AE) and is considered an emerging pathogen in some parts of the world (Eckert *et al*., 2000; Davidson *et al*., 2012). The life cycle of this parasite is completed in a two-host predator–prey system, which includes small mammals as intermediate host (mostly rodents, e.g. voles) and wild canids such as foxes (*Vulpes* spp.) and coyotes (*Canis latrans*), but also domestic dogs, as definitive hosts. Humans can get infected and develop AE by accidental ingestion of eggs (Thompson, 2017). Recently, the European strain of *E. multilocularis* was detected in Canada in wild, domestic, and human hosts, indicating a possible invasion of this strain in Western Canada, and the emergence of a new endemism (Jenkins *et al*., 2012; Gesy *et al*., 2013; Massolo *et al*., 2019; Santa *et al*., 2021). Using the invasion process of this strain as a model, we analysed the invasion of parasites with complex life cycles (CLC) transmitted in predator–prey systems, integrating concepts used in EIDs and biological invasion research. Since the life cycle of this parasite requires multiple hosts, it must overcome several eco-physiological barriers to colonize new areas and new host communities. In this review, the ecological and evolutionary factors that play a significant role during the process of invasion of these parasites were analysed, considering both host–parasite and host–host interactions, as well as the effects on the native parasite gene pool, the competition between parasite strains and the possible impact on host–parasite and predator–prey interactions.

## Ecological and evolutionary factors influencing parasite invasions

### The invasion process and its stages

The criteria to identify an invasive species imply multiple factors, based on taxonomy, biogeography, causes (natural *vs.* human-mediated), intensity and extent of the impact, and dynamics of invasion. Usually, an invasive alien species is defined as one that has been transported beyond the limits of its native range and has established a population in an area where it was not known to occur previously, resulting in negative impacts in the new environment (Lockwood *et al*., 2013). However, there is still some debate about the criteria to identify an invasive species. The discussion centres not only on the implications of a measurable impact (Ricciardi and Cohen, 2007), but also on the potential for native species to become invasive and the role of humans in mediating the invasions (Nackley *et al*., 2017). Colautti and MacIsaac (2004) proposed a conceptual framework based on the stages of the invasion process using neutral terminology to understand it as a biogeographical process of specific populations, rather than a taxonomic phenomenon. Likewise, Blackburn *et al*. (2011) proposed a framework for biological invasions using a more holistic approach, integrating into a single model concept used in invasions mediated by humans, regardless of taxon or location, including management intervention strategies at different stages of the process. Thus, four stages/phases of the process of invasion have been proposed: (1) translocation or transport, (2) introduction, (3) establishment, and (4) invasive spread (Kolar and Lodge, 2001). Therefore, an invasive species will be one that thrives and spreads widely, becoming dominant, with the potential to cause negative impacts in the colonized area.

During each stage of the invasion process, factors such as propagule pressure, biotic and abiotic conditions and community interactions are determinant for the success of invasion and may positively or negatively affect the spread of the invasive species (Colautti and MacIsaac, 2004; Blackburn *et al*., 2011). In the case of emergent diseases, parasites are usually translocated with their hosts and spilled over to native host communities following a similar progression of stages of invasion (Dunn and Hatcher, 2015). However, for parasites with CLC transmitted in predator–prey systems, interactions within food webs and the presence of multiple intermediate and definitive hosts become important in determining the survival and spread of the parasite. Therefore, although the phases of biological invasion and disease emergence have many parallels, host–host and host–parasite interactions and their co-evolution history play an important role in the establishment and spread of the parasite (Hatcher *et al*., 2012*b*). The study of the mechanism driving new host–pathogen associations and of the different factors that can play a role before and during the invasion process, are essential to predict and counteract possible negative impacts of parasite invasions.

### Drivers of parasite invasions and disease emergence

Climate change, habitat loss and fragmentation, changes in the use of water and land resources, and socio-economic activities can all be drivers for parasite invasions and disease emergence (Altizer *et al*., 2013; Hoberg and Brooks, 2015). Climate change, for example, has enabled species to expand and settle in regions where they previously could not survive (Walther *et al*., 2009). However, the identification of disease patterns has been challenging, particularly since climate change can limit the transmission of some pathogens, while creating opportunities for others (Altizer *et al*., 2013). For example, it has been suggested that high host specificity, CLC, and narrow climatic tolerance might increase the vulnerability of some parasites to climate change, particularly if there is some risk of coextinction with the host (Cizauskas *et al*., 2017). However, different outcomes can be expected depending on the species-specific eco-physiological responses of both hosts and parasites to environmental changes (Stensgaard *et al*., 2019). For example, for the trematode parasite *Schistosoma mansoni* and its intermediate freshwater snail host, risk-models predicted that some of north-eastern Africa might see a decline in transmission by more than 50% due to global warming (McCreesh *et al*., 2015). In contrast, in China, an expansion of *Schistosoma japonicum* into non-endemic areas in the northern part of the country has been predicted (Zhou *et al*., 2008), suggesting a shift rather than an expansion in the geographic distribution of schistosomiasis due to climate change (Stensgaard *et al*., 2019).

On the effect of climate change on parasites transmitted *via* direct *vs.* indirect life cycles, Molnár *et al*. (2013) suggested that behavioural thermoregulation by the intermediate host may protect parasites against extreme temperatures, generating a ‘shelter effect’, which would favour parasites with indirect cycles under future global warming conditions. Similarly, it has been hypothesized that for parasites with CLC, the prolonged survival of larval stages in intermediate hosts could extend the generation time over multiple seasons, potentially increasing the availability of infective propagules in the environment during the invasion process (Hoberg, 2010).

In addition to the effects of climate change on disease emergence, biological introductions due to transcontinental movements have brought to a global homogenization of flora and fauna, therefore influencing disease patterns globally (Young *et al*., 2017). Indeed, trade and transport are considered the most relevant drivers in biological invasions by microorganisms (Essl *et al*., 2020). For example, the change in the distribution of our model organisms (*E. multilocularis)* at a global level is associated with increased human travel, international trade and wildlife and/or domestic animal introductions (among other anthropogenic changes) which have allowed the spread of the parasite beyond historical endemic regions (Davidson *et al*., 2012). Similarly, for the parasite *Echinococcus granulosus* s.s., the causative agent of cystic echinococcosis, phylogenetic and phylogeographic analyses have demonstrated that the current widespread distribution and diversity of genotypes G1 (Kinkar *et al*., 2018*a*) and G3 (Kinkar *et al*., 2018*b*) have been shaped by intensive livestock trade, which has facilitated the dispersal of the parasite over vast geographic areas.

### Finding a new host: introduction and spillover

The introduction of alien parasites into new areas can produce new host(s)–parasite associations. Still, the effectiveness of spillover to new hosts will depend on the ability of the parasite to use new resources. Mostly, parasites are considered resource specialists with restricted host ranges, which has led to the idea that when parasites become specialized, it is at the expense of their ability to perform in alternative hosts (Agosta *et al*., 2010). However, host-switching seems to be commonly influencing the diversification of host–parasite associations (Araujo *et al*., 2015). The underlying mechanism of this process is termed ‘ecological fitting’ (Janzen, 1985), in which parasites can colonize new host species (with no co-evolutionary history) and create new associations, thanks to their phenotypic plasticity, and to the conservation of genetic information (phylogenetic conservatism) related to traits associated with host exploitation (i.e. resource use) (Agosta and Klemens, 2008). Consequently, these characteristics can facilitate invasion into new habitats and give the potential parasite fitness outside its natural range. The giant liver fluke, *Fascioloides magna,* for example, which originally cycled between North American ungulates (e.g. *Rangifer tarandus*, *Cervus canadensis*) as definitive hosts and freshwater snails as intermediate ones, was accidentally introduced to Europe. The parasite successfully found new intermediate and definitive hosts in native European species of snails (e.g. *Galba trunculata*), and ungulates (e.g. *Dama dama*), representing an example of multiple ecological fitting (Malcicka *et al*., 2015). Likewise, the trematode *Dicrocoelium dendriticum*, native to Europe, was introduced to North America, encountering new hosts to complete its three-host life cycle involving terrestrial snails and ants as first and second intermediate hosts, and ruminants as definitive hosts (van Paridon *et al*., 2017). However, some degree of eco-physiological equivalence in both biotic and abiotic conditions between the native and invaded ranges (including similar hosts) was necessary for successful colonization (Malcicka *et al*., 2015). Thus, the required conditions for an invasion by a parasite trophically transmitted might be even more restrictive if multiple host-switching events are necessary to complete its life cycle. However, co-introductions with the original host might help to overcome this barrier. In this way, the original host can act as a reservoir of the parasite for native hosts in a source−sink system, allowing more time for new host–parasite adaptations (Sokurenko *et al*., 2006).

Typically, a new host should be an inferior option compared to the original host if the parasite is specifically adapted (Kaltz and Shykoff, 1998). However, host-switching can happen rapidly, and there are multiple examples of parasites having a higher fitness on allopatric than sympatric hosts. For example, the swim bladder nematode (*Anguillicola crassus*), a parasite of the Japanese eel (*Anguilla japonica*), was introduced into the United Kingdom, where successfully infected native European eels (*Anguilla anguilla*), causing infections with higher worm intensities and pathogenic effects than in Japanese eels (Kirk, 2003). A possible explanation for this phenomenon is that when alien parasites are introduced to a new area, naïve hosts, which lack co-evolved resistance or tolerance, can suffer more significant pathogenic effects than co-evolved hosts (Allison, 1982). In a review of host–parasite co-invasions, in 85% of the cases, the **virulence** of the introduced parasite was higher in the new host than in the introduced host (Lymbery *et al*., 2014). However, high virulence is not always directly correlated with high parasite population fitness (i.e. capacity to survive and persist) since this might be enhanced either by increased or decreased virulence, depending on the characteristics of transmission (Cressler *et al*., 2016). While in early stages of invasion, higher virulence may be advantageous to the parasite, also increases selection pressure on the parasite towards a level that does not compromise long-term transmissibility (Anderson and May, 1982). Thus, the outcome of host–parasite interactions in the invasion process may not always be predictable, as different levels of virulence might be expected in an unusual host (Ebert, 1995). For example, differences in the co-evolutionary outcomes (resistance *vs.* tolerance) between the bivalve host *Mytilus edulis* and the invasive parasitic copepod *Mytilicola intestinalis* were observed in two different fronts of invasions in the North Sea, which was possibly related to local environmental differences (Feis *et al*., 2016). Therefore, spatial and temporal heterogeneity of the environment can shape the outcome of new host–parasite interactions and the process of invasion, affecting not only parasite infectivity and virulence, but also host immune responses.

### Biodiversity-parasite transmission relationship and invasion dynamics

Ecosystem traits along with host community diversity and composition, are important factors influencing successful invasions and parasite transmission in new environments. However, in the last years, there has been an intense debate on whether high-diversity systems reduce or increase the risk of transmission of infectious diseases, if there is a context-dependent relationship, or if there is no direct correlation (Rohr *et al*., 2020). The dilution effect hypothesis proposes that diverse ecological communities can limit pathogen spread, and so reduce disease risk (Johnson and Thieltges, 2010; Ostfeld and Keesing, 2012). Therefore, biodiversity loss could increase the transmission of parasites by indirectly or directly regulating populations of competent hosts, or changing the behaviour of the host, parasite, or vector (Keesing *et al*., 2010). However, the strength of the diversity−disease relationship varies depending on the parasite, and not all parasites are affected by changes in biodiversity (Rohr *et al*., 2020). In particular, CLC parasites with intermediate hosts or vectors and free-living stages would likely exhibit strong responses to changes in community diversity and composition, since multiple biological mechanisms influence the transmission process (Johnson and Thieltges, 2010; Rohr *et al*., 2020). Moreover, the response to these changes (increasing or decreasing disease risk) might depend on the host species composition rather than diversity *per se*. Therefore, the capacity to amplify or dilute parasite burden would differ depending on each species and their susceptibility, abundance and transmission potential (Levi *et al*., 2016), as well as the mode of transmission of the parasite (frequency- or density-dependent) (Young *et al*., 2017). For example, in the context of invasions, the Pacific oyster (*Crassostrea gigas*), introduced in the European Wadden Sea, can be infected by the native trematode *Renicola roscovita*; still, it is not consumed by native birds (definitive hosts). Thus, the Pacific oyster acts as a dead-end intermediate host, reducing the transmission of the parasite (Krakau *et al*., 2006). However, in the case of co-introductions, when new species adds more competent individuals to an unsaturated community, amplification of parasite transmission is expected since more competent host are available (Rohr *et al*., 2020). In the case of *E. multilocularis*, changes in community composition of the intermediate host have been associated with a higher prevalence of the parasite, when there is an increase in the relative abundance of competent hosts as a result of anthropogenic landscape disturbances (Giraudoux *et al*., 2003; Liccioli *et al*., 2015*b*). Since many parasites are able to infect phylogenetically close host species, thus, the phylogenetic and ecological structure of the receptive community needs to be considered to understand the diversity−parasite transmission relationship (Parker *et al*., 2015; Young *et al*., 2017).

### Effects of the parasite on host ecological interactions during the invasion process

Parasites can have a strong impact on host ecological interactions at all trophic levels and can play a significant role in co-invasions with their host (Prenter *et al*., 2004; Hatcher *et al*., 2006; Dunn *et al*., 2012). Such interactions include competition, predation, intraguild predation or ecological processes in which host species only interact *via* the indirect effects of the parasite (i.e. apparent competition). Through these interactions, the parasite can directly or indirectly affect host survival (population density-mediated effect) or host behaviour and life history (trait-mediated effect) (Hatcher et al., 2006). During the invasion process, parasite-mediated competition can result from the differential effects of the parasite on the fitness of competing host species (introduced *vs.* native), altering their ability to compete and ultimately affecting the outcome of invasion (Hudson and Greenman, 1998). Examples of disease-mediated invasion can be found across multiple taxa, including all kinds of parasites (macro- and microparasites, parasitoids and soil pathogens) and hosts (vertebrates, invertebrates and plants) (reviewed in Strauss *et al*., 2012). A classic example of disease-mediated invasion is the competitive exclusion of the populations of native red squirrel (*Sciurus vulgaris*) in the UK, mediated through the transmission of a *Parapoxvirus* that was introduced with the arrival of the North American grey squirrel (*Sciurus carolinensis*) (Tompkins *et al*., 2002). Likewise, the meningeal worm (*Parelaphostrongylus tenuis*) transmitted through snails, facilitated the range expansion of North American white-tailed deer (*Odocoileus virginianus*), leading to dramatic decreases of moose (*Alces alces*) and caribou (*R. tarandus*) populations in the north-eastern United States in the mid-1900s (Anderson, 1972).

Parasites transmitted through predator–prey systems can also alter host interactions. For example, parasites can manipulate the behaviour of the intermediate host (prey), increasing its susceptibility to predation and thereby the probability of the parasite reaching the definitive host (Moore, 2002; Poulin and Maure, 2015). Although some changes in host behaviour can be non-adaptive by-products of infection, host manipulation by parasites is a common adaptive strategy of parasites (Poulin and Maure, 2015). In the case of *E. multilocularis*, there is no direct evidence of increased vulnerability to predation in infected intermediate hosts. However, mathematical models have shown that increased susceptibility to predation enhances parasite persistence even at extremely low predator densities, which could be related to the observed prevalence rebounding after anthelmintic treatment in foxes as part of Echinococcosis control programmes (Vervaeke *et al*., 2006). During the invasion process, host manipulation could increase parasite transmission, depending on the ability of the parasite to manipulate different intermediate hosts. For example, the invasive American brine shrimp, *Artemia franciscana,* and the native shrimp, *A. parthenogenetica* from the Mediterranean region, share a cestode parasite that causes reverse phototaxis and colour change in the native, but not in the invasive brine shrimp, leading to higher predation of the native shrimp by bird definitive hosts (Georgiev *et al*., 2007). Thus, the differential effect of the parasite in the new and original host has the potential to alter the characteristics of food webs, to influence types of interactions in ecological networks, affecting community structure and ecosystem stability in the invaded location (Hatcher *et al*., 2012*a*; Jephcott *et al*., 2016).

### Idiosyncratic characteristics of the invasion process: founder events and propagule pressure

During the process of invasion, the likelihood of successful establishment will strongly depend on the propagule pressure, which refers to the number of introduction events and the number of infective stages released. Both observational and experimental analyses found that propagule pressure explains significant variation in the outcome of biological invasions across different taxa and locations (Lockwood *et al*., 2005). Therefore, the repeated release of a large number of individuals in multiple locations helps to overcome problems of small population size, facilitating long-term establishment. For example, during founder events, introduced small populations can suffer genetic bottlenecks and increased inbreeding levels that reduce adaptive potential compared with larger populations resulting from multiple introductions. Hence, multiple introductions help increase the genetic variability and improve the ability of introduced populations to adapt to the novel selection pressures (Lockwood *et al*., 2005; Roman and Darling, 2007). However, multiple introductions and high genetic variation do not seem to be indispensable for a successful invasion, and even genetically impoverished populations have the potential to evolve rapidly. In a review addressing the link between genetic diversity and invasion success, Dlugosch *et al*. (2015) found that genetic variation rarely shapes the invasion process. They proposed that the effect of genotypes on phenotype expression and the kind of genetic variation that is introduced, rather than the quantity, might play a more definitive role. Moreover, low variability in single or few markers is usually not an adequate measure of the species capacity to adapt to new environmental conditions.

The introduction, establishment, and invasive spread of species, despite potentially costly genetic bottlenecks, represent a genetic paradox. However, factors such as reproductive traits of most parasites (self-fertilization or high reproductive output), may prevent or increase tolerance to genetic depletion and inbreeding depression during the invasion process (Frankham, 2004). For a parasite like *E. multilocularis*, reproductive traits could be an advantage in the first stages of invasion. They are hermaphrodite, capable of self- and cross-fertilization, and have a phase of asexual multiplication that produces a large number of protoscoleces, developing thousands of sexually mature adult worms. However, although genetic admixture and reproductive traits have been proposed to explain this genetic paradox, the mechanisms behind successful invasions, despite substantial genetic depletion, inbreeding depression, and drift loads, have not been completed elucidated (Schrieber and Lachmuth, 2017).

### Within-host parasite interactions and their effects on the local parasite population

The infection of individual hosts by multiple parasite strains or species is common in nature, generating competitive or cooperative interactions between parasites. These parasite interactions represent a primary evolutionary force shaping parasite survival, growth, reproduction, and transmission (Mideo, 2009), thus influencing the colonization of new host species

Intra- and inter-specific parasite competition would depend on resource availability (exploitation competition), immune response (apparent competition), or direct interference (Read and Taylor, 2001; Lello *et al*., 2004). For example, the abundance of within-host resources can be a limiting factor, leading to selection for divergence in resource use or adaptation to increase the ability to exploit the shared resource (Mideo, 2009). Therefore, multiple infections can promote the evolution of high virulence due to faster depletion of host resources (Alizon *et al*., 2013). Although this has been predicted in mathematical models and observed in lab experiments (reviewed in Cressler *et al*., 2016), higher virulence is not always found in the wild, and the genetic relatedness of coinfecting parasites may play a more important role in shaping the evolution of virulence (Read and Taylor, 2001; Alizon *et al*., 2013). Moreover, recent evidence suggests that interactions among co-infecting parasites are key for maintaining genetic variation in parasite traits such as infectivity and virulence, thereby influencing the co-evolutionary dynamics between hosts and parasites (Seppälä and Jokela, 2016).

In the context of invasion, when previously allopatric lineages come into contact and interbreed, novel allelic combinations can be generated with positive and negative effects for the invasive and native parasite population (Shi *et al*., 2018). Potential benefits for the invasive parasite include heterosis, decreasing inbreeding depression, and enhancing adaptive potential (Roman and Darling, 2007; Rius and Darling, 2014). Additionally, the emergence of novel genotypes can also have an important role in providing opportunities for local adaptation (Verhoeven *et al*., 2011; Rius and Darling, 2014). Yet, the introduction of novel genotypes can also cause outbreeding depression, producing a ‘dilution’ of locally adapted genotypes with a subsequent increase of maladaptive genotypes (Verhoeven *et al*., 2011). However, the co-occurrence of divergent lineages does not necessarily involve genetic admixture (Rius and Darling, 2014). Intraspecific competition between these related strains also occurs and is likely to be more intense than between different species of parasites, due to overlapping ecological niches and possible immune cross-reactions (Read and Taylor, 2001; Alizon *et al*., 2013). The effect of this competition was tested by experimentally infecting laboratory mice with the protozoan parasite, *Trypanosoma brucei* (causal agent of human African sleeping sickness), using strains with different levels of virulence. The experiment showed a strong mutual competitive suppression of co-infecting strains in early stages of infection, resulting in decreasing effects of infection in the host (Balmer *et al*., 2009). On the other hand, high relatedness between co-infecting genotypes has also been proposed as a factor that can promote cooperation between parasites, resulting in an indirect increase in their fitness (Leggett *et al*., 2014). For *E. multilocularis*, mixed infections of different *Echinococcus* and *Taenia* species in individual definitive hosts have been commonly reported (Knapp *et al*., 2009; Liccioli *et al*., 2012; Umhang *et al*., 2017; Massolo *et al*., 2018; Santa *et al*., 2018). Similarly, mixed infections of *E. multilocularis* genetic variants are relatively common (Umhang *et al*., 2017; Santa *et al*., 2021), but the heterozygosity observed in some studies was low, suggesting a rare occurrence of outcrossing (Nakao *et al*., 2003; Knapp *et al*., 2008). Thus, self-fertilization could be common during the first stages of invasion when a small number of propagule founders is present (Lymbery, 2017). Investigating the differences in virulence and infectivity and its effect on the intraspecific competition between multiple *E. multilocularis* strains would help to understand their differential invasiveness potential.

After having analysed the ecological and evolutionary factors that can play a significant role during the invasion process of CLC parasites, it is clear that the influence of multiple factors on within-host interactions – including host-immune response, parasite life-traits, population density effects, phenotypic plasticity, and genotype-environmental interactions – can all change the outcome of different invasion events by the same parasite. Thus, the analysis of current processes of invasions of parasites transmitted in cycles involving multiple hosts would help understand common pathways and potential effects of similar parasite invasions.

## The invasion of the European strain of *Echinococcus multilocularis* into North America

### Translocation and introduction of the European strain

There is increasing evidence that *E. multilocularis* is expanding both its geographic and host range, associated with changes in the distribution of its definitive hosts due to anthropogenic effects (Eckert *et al*., 2000; Giraudoux *et al*., 2003; Davidson *et al*., 2012). In North America, *E. multilocularis* was first reported in the Northern Tundra Zone (NTZ) of Alaska and the Canadian Arctic. Only after the 1960s, the parasite was reported in the Northern Central region (NCR), which include 13 contiguous states of the USA and the southern area of three Canadian provinces (Alberta, Saskatchewan and Manitoba) (Eckert *et al*., 2000). However, recent studies have provided evidence of the parasite presence in previously non-endemic regions in Canada such as British Columbia (BC) (Gesy *et al*., 2013), Southern Ontario (Kotwa *et al*., 2019), and the taiga region between NTZ and NCR (Schurer *et al*., 2016).

**Fig. 1.**
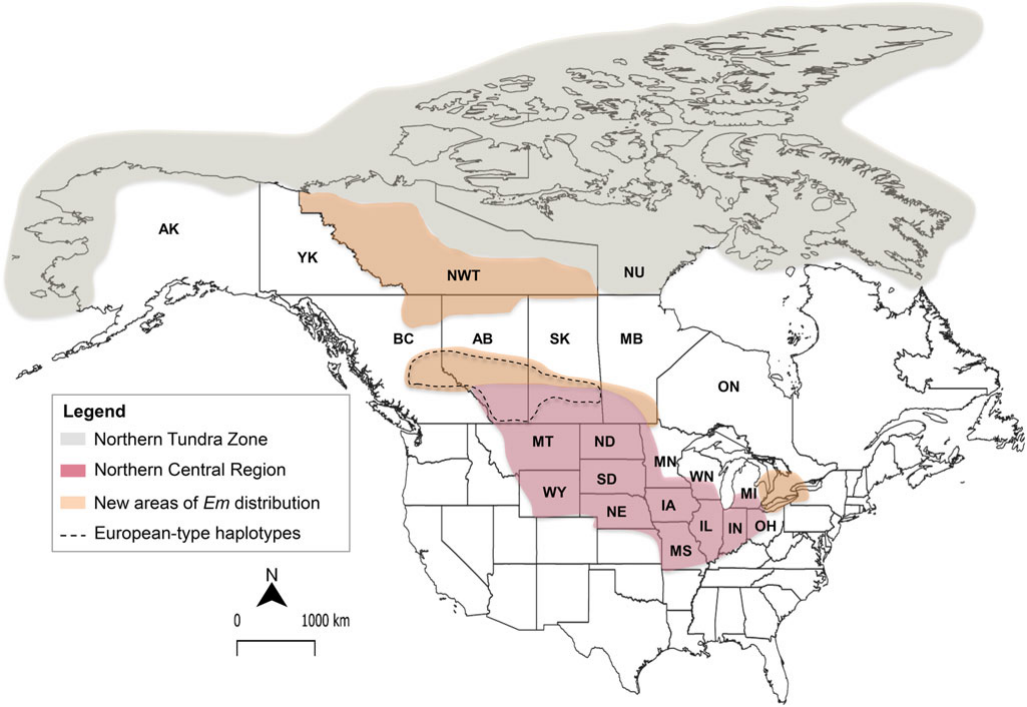
Distribution of European-type haplotypes of *E. multilocularis* in North America. This map shows the historical area of distribution of *E. multilocularis* (*Em*) corresponding to the Northern Tundra Zone (NTZ) and Northern Central Region (NCR), the new endemic regions, and the locations in which European-type haplotypes have been identified (based on *cox1*, *cob* and *nad2* genes). Abbreviations: Alaska (AK), Yukon territory (YT), Northwest Territories (NT), Nunavut (NU), British Columbia (BC), Alberta (AB), Saskatchewan (SK), Manitoba (MB), Montana (MT), Wyoming (WY), North Dakota (ND), South Dakota (SD), Nebraska (NE), Minnesota (MN), Iowa (IA), Wisconsin (WI), Illinois (IL), Michigan (MI), Indiana (IN) and Ohio (OH). References: (Eckert *et al*., 2001; Nakao *et al*., 2009; Jenkins *et al*., 2012; Gesy *et al*., 2013; Schurer *et al*., 2013, 2016; Gesy and Jenkins, 2015; Santa *et al*., 2021).

Despite a significant increase in epidemiological research in the last years, information about the genetic diversity of *E. multilocularis* in North America is still largely unknown. Nakao *et al*. (2009) assessed the intraspecific genetic diversity of the parasite describing four genetic strains worldwide (Asian, European, North American and Mongolian), which are believed to were isolated during repeated glacial events during the Pleistocene. In Canada and the United States, two haplotypes of the North American strain (N1, N2) were described, each one associated with the NTZ and NCR areas. However, recent studies have revealed the circulation of European-type haplotypes in wild canids in British Columbia, Saskatchewan, and Alberta (Gesy *et al*., 2013; Gesy and Jenkins, 2015; Massolo *et al*., 2019) (Fig. 1), and multiple aberrant cases of AE in dogs in previously non-endemic areas of British Columbia (Jenkins *et al*., 2012) and Ontario (Skelding *et al*., 2014; Oscos-Snowball *et al*., 2015). Moreover, there has been an unprecedented outbreak of human AE cases, with 17 locally acquired cases described since 2013 in the province of Alberta (Massolo *et al*., 2019; Houston *et al*., 2021) and the first confirmed human case in Saskatchewan (Schurer *et al*., 2020), after only two locally acquired cases ever reported (in 1923 and 1977) for continental North America (Massolo *et al*., 2014; Klein and Massolo, 2015). Furthermore, molecular characterization (when possible) confirmed the European strain as the causative agent, yet none of these patients had travelled outside Canada, suggesting autochthonous transmission of this strain (Massolo *et al*., 2019).

Genetic characterization and phylogenetic analysis of the European-type haplotypes in Western Canada have shown a close relationship with original European clades (Gesy *et al*., 2013; Gesy and Jenkins, 2015; Massolo *et al*., 2019), which supports the hypothesis of a relatively recent introduction, facilitated through translocation of domestic dogs (Jenkins *et al*., 2012), *via* intermediate hosts translocated with international shipping (Davidson *et al*., 2012), and/or introduced red foxes (*Vulpes vulpes*) imported for sport hunting from France during the last century (Kamler and Ballard, 2002). Another possible indicator of a recent invasion is the increasing reports of unusual clinical manifestations of AE in dogs, with severe and often lethal infections (Jenkins *et al*., 2012; Peregrine, 2015). This could be related to a general increase in the exposure to the parasite (Deplazes and Eckert, 2001), for example, in urban areas (Liccioli *et al*., 2015*b*), and the presence of introduced parasite strains with high pathogenic potential, such as the European strain (Nakao *et al*., 2009). In Canada, there are no strict screening requirements for *E. multilocularis* for pets imported into the country, and there are no regulations for the relocation of dogs within and between provinces (Canadian Food Inspection Agency, 2018). Between 2013 and 2014 alone, 6200 dogs were imported into Canada from 29 countries, including from Europe (Anderson *et al*., 2016; Julien *et al*., 2021), which poses a high risk of introduction of the parasite. Indeed, a risk assessment of importation of dogs from endemic countries infected with *E. multilocularis* into the UK found that, without the mandatory treatment with praziquantel, the probability of at least one infected dog returning to the UK was approximately 98% (Torgerson and Craig, 2009). On the other hand, an introduction *via* intermediate hosts is less likely due to the generally low prevalence of the parasite in these hosts (Romig *et al*., 2017). In the UK, for example, *E. multilocularis* was found in a captive beaver imported from Germany (Barlow *et al*., 2011); however, despite the potential risk of introduction, there have been no known domestically acquired cases in the UK.

Another possible mechanism of introduction to consider could be through the dispersal movements of arctic foxes (*Vulpes lagopus*), as evidenced by the satellite tracking of natal dispersal by a young female between continents, from Svalbard Archipelago (Norway) to Ellesmere Island, Nunavut (Canada), in 76 days (Fuglei and Tarroux, 2019). However, only the Asian and North American strains have been reported in the northern territories in North America, whereas the European strain seems to be restricted to the NCR (Fig. 1) (Nakao *et al*., 2009; Gesy *et al*., 2013; Gesy and Jenkins, 2015; Massolo *et al*., 2019; Santa *et al*., 2021). Therefore, introduction with red foxes and/or dogs, seems to be the more plausible source of invasion, with multiple introduction events having occurred during the last century, as evidenced by the limited distribution of some European-type haplotypes in each western province (Santa *et al*., 2021).

### Establishment into local predator–prey systems

In the core endemic area of Europe red foxes appear to be responsible for most of the environmental contamination with *E. multilocularis* eggs (Eckert and Deplazes, 1999), being the primary host of the parasite, which at the same time implies a long co-evolutionary history. Likewise, it was assumed that the red fox was the primary host in the NCR in North America. However, the coyote (*C. latrans*) is likely another important definitive host in that area, due to its larger home range, dispersal distances and increasing presence in urban environments (Catalano *et al*., 2012; Gesy *et al*., 2013; Liccioli *et al*., 2015*b*; Deplazes *et al*., 2017). Moreover, interspecific competition between coyotes and red foxes has decreased red fox populations densities, since coyotes seem to displace them and could even prey upon them (Gosselink *et al*., 2003; Liccioli *et al*., 2015*a*), which may generate in turn, changes in definitive host communities and shift in the role of the different hosts in the transmission of the parasite (Liccioli *et al*., 2014).

After its introduction in North America, the European strain may have benefited from the most abundant definitive host available, the coyote. The abundance and distribution of this host species across North America have increased due, in part, to the agricultural expansion after European colonization, which created an ideal habitat for them (Gompper, 2002). Thus, coyotes, being naïve hosts, lacking co-evolved resistance or tolerance to the European strain, may have played a significant role in the environmental contamination with eggs by this strain. Although the dietary preferences of coyotes vary across their range, small mammals are a staple for coyotes in urban environments, and up to 80% of these prey species are competent hosts for *Em* (Liccioli *et al*., 2015*a*). Moreover, the prevalence of *Em* in coyotes was reported being up to 83.8% in a hyperendemic area in the city of Calgary (Liccioli *et al*., 2014), and, on average, 24% in urban and rural environments in Alberta (Catalano *et al*., 2012), which supports the key role of coyotes in the transmission of the parasite.

In the endemic area of *Em* in Central Europe, intermediate hosts are mainly voles, *Microtus arvalis,* and *Arvicola* spp. (Romig *et al*., 2017). In the NCR in North America, the European strain found competent intermediate hosts in meadow voles (*Microtus pennsylvanicus*) and deer mice (*Peromyscus maniculatus*), but also newly described hosts such as the southern red-backed vole (*Myodes gapperi*) (Liccioli *et al*., 2013). The establishment of the European strain may have also affected the population dynamics of intermediate host communities through apparent competition or host manipulation, potentially altering the predator–prey relationships and the *E. multilocularis* transmission. Differences in metacestode development and susceptibility to infection in intermediate hosts have been observed between parasite isolates from different geographical regions (Rausch and Richards, 1971; Bartel *et al*., 1992). In experimental infections, meadow voles were all susceptible to isolates from North America (St. Lawrence Island) and Europe (Germany) and developed fertile metacestodes (Obayashi *et al*., 1971). On the other hand, Rausch and Richards (1971) observed that metacestodes in deer mice contained fewer protoscoleces than meadow voles. Moreover, coyotes were found to select against deer mice, in favour of voles, despite a relatively higher abundance of deer mice in urban areas (Liccioli *et al*., 2015*a*). Therefore, even if the average prevalence of *Em* in deer mice is similar to meadow voles (Liccioli *et al*., 2015*a*), susceptibility to different parasite strains and predator–prey relationships could influence the transmission of the parasite. However, the specific pathogenicity and zoonotic potential of the European strain have not been empirically tested or compared to other strains, and overall, the individual role of different players in this multi-host transmission system has not yet been elucidated.

### The spread of the European strain and competition with native strains

**Fig. 2.**
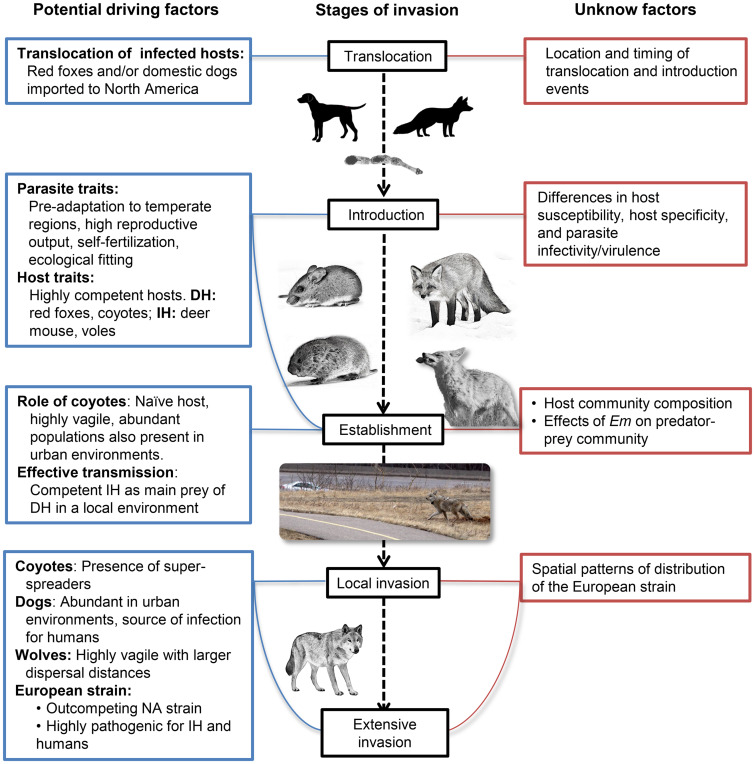
Suggested process of invasion of the European strain of *Echinococcus multilocularis* in North America. The European strain successfully overcame geographic, ecological, and evolutionary barriers, allowing its widespread in Western Canada. Although multiple driving factors for the invasion have been identified during its translocation, introduction, establishment and spread stages, there are still unknown factors to explain the extent of the invasion of this strain. IH: Intermediate host, DH: Definitive host. *Em: Echinococcus multilocularis.*

Genetic studies analysing mitochondrial and nuclear DNA of *E. multilocularis*, have found a distant genetic relationship between *E. multilocularis* isolates from Europe and North America, when comparing isolates from distinct geographical regions (Bowles *et al*., 1992; Nakao *et al*., 2009; Knapp *et al*., 2010). Only until 2009, a haplotype closely related to a European strain from west-central Europe (E4), was detected in a dog in British Columbia, Canada (Jenkins *et al*., 2012), leading to increased efforts in identifying the extent of the invasion of the European strain into North America. A subsequent survey in coyotes and foxes within the same area in British Columbia confirmed that the European-type haplotype (BC1) was the only one detected in this new endemic region in definitive hosts (Gesy *et al*., 2013). A different European-type haplotype (SK1) was found in coyotes from the peripheral area of Saskatoon, Saskatchewan, while, in the southwest of the province, six haplotypes similar to the North American (N2) strain were found in deer mice (Gesy and Jenkins, 2015). The situation in Alberta was even more surprising since all of the identified haplotypes were closely related to the European strain (except from one sample) (Gesy *et al*., 2014; Massolo *et al*., 2019; Santa *et al*., 2021), despite it being a historically endemic region for the NA strain. These results suggest that the European strain is widespread within Western Canada, forming complex mosaics with the North American strain, and potentially out-competing it. After introduction and establishment, the spread of the European strain could have been boosted by the presence of highly vagile, abundant and susceptible hosts like coyotes. Additionally, wolves (*Canis lupus*) were recently confirmed as regular definitive host of *E. multilocularis* (Schurer *et al*., 2013, 2016; Gesy *et al*., 2014), potentially contributing to a broader spread of the strain into northern areas, due to their large home range (>62 000 km^2^ in the artic) and dispersal distances (50–800 km) (Walton *et al*., 2001) (Fig. 2).

In the NCR, the European strain was found in AE cases of dogs with no travel history (Kotwa *et al*., 2019). Moreover, in the city of Calgary, a study found one out of 218 dog faecal samples to be positive to *Em* after molecular confirmation (Massolo *et al*., 2014), whereas in a survey across the Canadian provinces, the parasite was not detected in 1608 faecal samples from shelter dogs (Villeneuve *et al*., 2015). Since limited information is available about the true prevalence of intestinal infection, the definitive role of domestic dogs in the transmission of *E. multilocularis*, and the genetic variants circulating in dog populations in North America still need to be determined (Massolo *et al*., 2014; Toews *et al*., 2021). Certainly, dogs are highly susceptible to *Em* infection and the parasite has a high biotic potential in this host (Kapel *et al*., 2006). For example, in highly endemic areas of central Asia, free-roaming dogs have shown a prevalence of up to 26% (Ziadinov *et al*., 2008). Therefore, domestic dogs might play a pivotal role in the transmission of the European strain in North America, potentially acting as the main source of infection in humans as suggested by recent findings (Massolo *et al*., 2019) due to their large population in urban areas (e.g. >125 000 licensed dogs in 2013 in Calgary, AB) and increased exposure to *E. multilocularis* linked to the presence of coyotes in urban areas.

## The missing link: an ecological framework for CLC parasites transmitted in predator–prey systems

Parasitic diseases caused by parasites with CLC come from a wide range of taxonomic groups, yet they share many features in their life histories. From an evolutionary perspective, incorporating an intermediate host can maximize parasite fitness by increasing the number of surviving progeny in the next generation (Chubb *et al*., 2010). In predator–prey systems, intermediate hosts are species that occupy key positions in food webs, thus facilitating transmission of the parasite (Poulin and Maure, 2015). Infecting predators that prey upon multiple individuals in a relatively short time is also an efficient way for parasites to meet a sexual partner increasing the opportunities for cross-fertilization (Brown *et al*., 2001). Moreover, reaching long-lived and highly vagile definitive hosts such as large carnivores promotes higher growth (with consequently high fecundity), longevity and broad spatial dispersion (Ewald, 1995; Parker *et al*., 2003). Therefore, for a parasite transmitted in prey–predator systems, all these characteristics can give an advantage for the successful colonization of new environments and hosts, helping to increase spatial distribution across heterogeneous environments and over extended time frames (Hoberg, 2010). Nonetheless, the complexity of the transmission process may also prevent substantial changes in the parasite distribution and host range (Cleaveland *et al*., 2001). If suitable hosts for all parasite life cycle stages are not present, or the parasite cycle becomes truncated in dead-end hosts, the parasite will not become established (Torchin *et al*., 2003). This problem may be overcome in the case of co-introductions of parasites and their hosts. For example, the local establishment of *E. multilocularis* into the Svalbard Archipelago in the Norwegian Arctic was enabled by the co-introduction of the sibling vole (*Microtus rossiaemeridionalis*), since no suitable intermediate host was previously present (Henttonen *et al*., 2001).

Many alien parasites are co-introduced with an alien host species, and the successful establishment of parasites with CLC is not unusual. A review by Lymbery *et al*. (2014) found that, of 98 studies on co-introductions of different taxa, 36% involved parasites with an indirect life cycle. However, when considering the impact of the invasive parasite species, only one species (*Plasmodium relicta*) out of the eight parasites included in the IUCN list of worst invasive alien species has an indirect cycle (Hatcher *et al*., 2012*b*). Hence, most harmful emergent diseases, impacting human and animal health, are transmitted through direct cycles (Jones *et al*., 2008; Tompkins *et al*., 2015). However, this could instead reflect more significant research and surveillance efforts towards emerging diseases affecting humans and domestic animals, which calls for more efforts to understand the actual extent of invasions for parasites with CLC involving wildlife. Under current climate change, CLC parasites transmitted in predator–prey systems may have an advantage under extreme temperatures and greatest ability to disperse, becoming an important source of future emergence diseases.

**Fig. 3.**
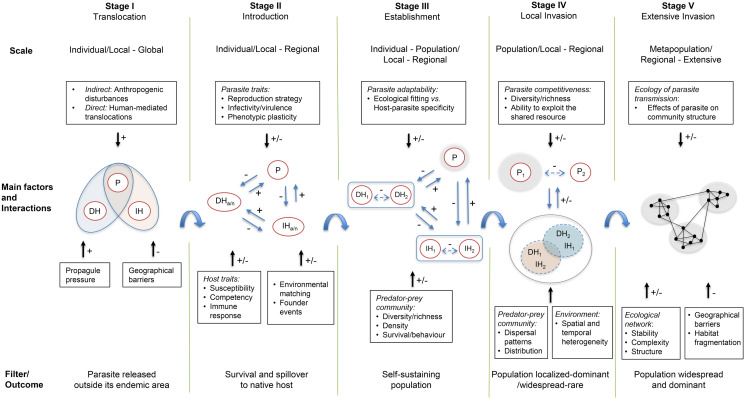
Framework for the invasion process of parasites trophically transmitted in predator–prey systems. During each stage, biological processes are occurring at different time, space and ecological scales. These processes depend upon intrinsic characteristics of the predator–prey system and the interactions between biotic and abiotic components. This framework shows the main factors that are determinant during the invasion process and may affect the host–parasite interactions within the predator–prey community and the transition to subsequent stages of invasion. The + and − symbols indicate positive and negative effects. Solid and broken lines represent direct and indirect effects, respectively. P: Parasite, DH: Definitive host, IH: Intermediate host. Lower case letters ‘a’ and ‘n’ stand for alien and native species.

In the previous sections of this review, we identified ecological and evolutionary factors that could influence the invasion process of parasites with CLC. Based on the conceptual invasion frameworks developed by Colautti and MacIsaac (2004) and Blackburn *et al*. (2011), and the process of invasion of the European strain of *Em* in North America, we propose a framework (Fig. 3) to summarize the mechanisms previously identified, integrating concepts used in EIDs and biological invasion research to understand the invasion process of parasites transmitted in predator–prey systems from an eco-evolutionary perspective. We used the model of ‘stages’ and ‘filters’ that the potential invader must overcome, and included key processes occurring at each stage of invasion, the role of the interactions between the host−parasite−environment and the differences in the temporal-space scale. In the first stage of invasion, the translocation of the parasite can be mediated by indirect factors such as anthropogenic disturbances that generate in turn changes in the distribution of the hosts; or can be directly human-mediated through the transport of propagules, or the movement of the original hosts along with the parasite, associated with local/global trade and transport (Hatcher *et al*., 2012*b*; Altizer *et al*., 2013). During this stage, the number of propagules translocated and geographical barriers that historically prevented natural dispersal are critical, facilitating or not the release of the parasite beyond the limits of its native range. In the second stage of invasion, the introduction and spillover to native hosts would depend on specific traits of the parasite (e.g. reproduction strategy, phenotypic plasticity), the characteristics of the local hosts (e.g. susceptibility, biotic potential), the number and frequency of infective stages introduced, if the parasite is co-introduced with its original hosts, and environmental matching, that would help to overcome biotic and abiotic barriers to survive and form new host–parasite associations (Frankham, 2004; Lockwood *et al*., 2005; Dunn and Hatcher, 2015). At the same time, these factors can positively or negatively affect interactions between the parasite, and the intermediate and definitive hosts (native/alien) within the predator–prey system. These interactions occur at an individual level, at local or regional geographic scale (depending on the number of founder events), with possible differential impacts on the native/alien host, which could facilitate or not the transmission of the parasite (Prenter *et al*., 2004; Hatcher *et al*., 2006; Lymbery *et al*., 2014). Therefore, during the introduction and establishment stages, the parasite must overcome survival and reproductive barriers to rapidly adapt to the new environment. In the third stage of invasion, the persistence of the parasite in the recipient area would depend on the pre-adaptation characteristics of the parasite *via* the eco-evolutionary experience in previous environments, allowing individuals to survive as an infective propagule, find and infect new intermediate and definitive hosts, and to reproduce, completing their life cycle with the establishment of a self-sustaining population in the long-term (Agosta and Klemens, 2008; Ogden *et al*., 2019). Additionally, the characteristics of the predator–prey community, such as diversity/richness and hosts population size and density, would influence the transmission of the parasite, amplifying or diluting parasite burden (Keesing *et al*., 2010; Young *et al*., 2017). Moreover, if the parasite is introduced with its original hosts, they could act as a reservoir for native hosts, allowing more time for new host–parasite adaptations (Sokurenko *et al*., 2006). At the same time, ecological interactions between different species of definitive and intermediate hosts within the predator–prey community might be influenced by the parasite, *via* direct or indirect effects on host behaviour and survival, thus, potentially promoting competition and predation and enhancing the transmission of the parasite (Hudson and Greenman, 1998; Hatcher *et al*., 2006; Dunn *et al*., 2012). At this stage, the parasite population is small and localized and numerically rare. However, in the fourth stage of invasion, the population can become either localized and dominant, or widespread but rare.

The local or regional dispersal, and dominance of the parasite over other species/strains, would depend on the within-host intra- and interspecific interactions and the ability to exploit the shared resource, as well as the dispersal patterns and distribution of the host populations (Mideo, 2009; Alizon *et al*., 2013). Furthermore, the spatial and temporal heterogeneity of the founder events and the environmental conditions can also shape the outcome of new host–parasite interactions and the process of invasion, limiting or promoting an extensive spread of the parasite. In the last stage of the invasion, the parasite population can become widespread and dominant after overcoming geographical and dispersal barriers to find new suitable host communities at multiple sites. This process would depend on the stability, complexity and structure of the ecological network in which the parasite is transmitted, and the potential effect of the parasite on the interactions within the food web, that will allow the spread to multiple predator–prey communities over extensive areas away from the point of introduction (Hatcher *et al*., 2012*b*; Jephcott *et al*., 2016). Thus, incorporating network analysis concepts is pivotal to understand the ecology and heterogeneity of parasite transmission and its potential invasive spread, especially for parasites that depend to some extent on the behaviour of the host to enable transmission. The application of social networks to study the epidemiology of wildlife parasites has been growing in the last years, especially for EIDs (Godfrey, 2013). However, a broader scope and incorporation of parasites transmitted in complex host–parasite systems is still needed.

## Concluding remarks

Biological invasions are multifactorial, complex phenomena that involve largely idiosyncratic ecological characteristics. Despite the vast body of knowledge about underlying processes influencing invasion dynamics, there is an incomplete understanding of the interaction between macro and micro-evolutionary processes and what drives the potential for invasion. Additionally, the research on parasitism in the context of biological invasions has advanced very slowly, compared to the study of biological invasions by animals or plants (Ogden *et al*., 2019). The capability of identifying the introduction of non-indigenous parasites is limited by the absence of comprehensive taxonomic inventories of parasites, including molecular characterization. Therefore, molecular-based surveillance is key to exploring the distribution and diversity of exotic and native species, and to be able to understand the evolutionary and biogeographic history of invasion processes.

Although parasites with CLC may have to overcome more ecological and physiological barriers to invade new environments successfully, there are examples of colonization in heterogeneous environments, including parasites with high impact on animal and human health (Henttonen *et al*., 2001; Malcicka *et al*., 2015; van Paridon *et al*., 2017). The analysis of the invasion process of the European strain of *E. multilocularis* in North America is particularly important to our understanding of the specific factors influencing the invasion process of those parasites that are trophically transmitted in predator–prey systems. Understanding the historical origins and complex components of these new host–parasite mosaics is essential in formulating predictions about future invasions, and elucidate the effects of climate change and ecological disturbance on the potential for invasion. Moreover, since most parasites are introduced with their original hosts, there is a need for integration between the study of biological invasions and disease ecology, which would allow the design of comprehensive predictive frameworks assessing the risk of invasion and lead to possible management strategies. This mandates for multidisciplinary studies on mechanisms, effects, and the control of parasite invasions, focusing not only on host–parasite interactions, but also on the broader impacts of these invasions at the ecosystem level.

## Supplementary material

For supplementary material accompanying this paper visit https://doi.org/10.1017/S0031182021001426. click here to view supplementary material

## References

[ref1] Agosta SJ and Klemens JA (2008) Ecological fitting by phenotypically flexible genotypes: implications for species associations, community assembly and evolution. Ecology Letters 11, 1123–1134. 18778274 10.1111/j.1461-0248.2008.01237.x

[ref2] Agosta SJ, Janz N and Brooks DR (2010) How specialists can be generalists: resolving the “parasite paradox” and implications for emerging infectious disease. Zoologia *(*Curitiba*)* 27, 151–162.

[ref3] Alizon S, de Roode JC and Michalakis Y (2013) Multiple infections and the evolution of virulence. Ecology Letters 16, 556–567. 23347009 10.1111/ele.12076

[ref4] Allison AC (1982) Co-evolution between hosts and infectious disease agents and its effects on virulence. In Anderson RM and May RM (eds). Population Biology of Infectious Diseases. Dahlem Workshop Reports, vol 25. Berlin: Springer, pp. 245–267.

[ref5] Altizer S, Ostfeld RS, Johnson PTJ, Kutz S and Harvell CD (2013) Climate change and infectious diseases: from evidence to a predictive framework. Science (New York, N.Y.) 341, 514–519. 10.1126/science.1239401 23908230

[ref6] Anderson RC (1972) The ecological relationships of meningeal worm and native cervids in North America. Journal of Wildlife Diseases 8, 304–310. 4564182 10.7589/0090-3558-8.4.304

[ref7] Anderson RM and May R (1982) Coevolution of hosts and parasites. Parasitology 85, 411–426. 6755367 10.1017/s0031182000055360

[ref8] Anderson M, Douma D, Kostiuk D, Filejski C, Rusk R, Weese JS, Bourque T, Lee-Fuller C and Rajzman C (2016) Report of the Canadian National Canine Importation Working Group. Retrieved 8 July 2021, Available at https://www.canadianveterinarians.net/documents/canadian-canine-importation-working-group-report.

[ref9] Araujo SB, Braga MP, Brooks DR, Agosta SJ, Hoberg EP, von Hartenthal FW and Boeger WA (2015) Understanding host-switching by ecological fitting. PLoS One 10, e0139225. 26431199 10.1371/journal.pone.0139225 PMC4592216

[ref10] Balmer O, Stearns SC, Schotzau A and Brun R (2009) Intraspecific competition between co-infecting parasite strains enhances host survival in African trypanosomes. Ecology 90, 3367–3378. 20120806 10.1890/08-2291.1

[ref11] Barlow AM, Gottstein B and Mueller N (2011) *Echinococcus multilocularis* in an imported captive European beaver (*Castor fiber*) in Great Britain. Veterinary Record 169, 339–339. 10.1136/vr.d4673 21900259

[ref12] Bartel MH, Seesee FM and Worley DE (1992) Comparison of Montana and Alaska isolates of *Echinococcus multilocularis* in gerbils with observations on the cyst growth, hook characteristics, and host response. The Journal of Parasitology 78, 529–532. 1597801

[ref13] Bellard C, Genovesi P and Jeschke JM (2016) Global patterns in threats to vertebrates by biological invasions. Proceedings of the Royal Society B: Biological Sciences 283, 20152454. 10.1098/rspb.2015.2454 PMC4795027 26817767

[ref14] Blackburn TM, Pyšek P, Bacher S, Carlton JT, Duncan RP, Jarošík V, Wilson JRU and Richardson DM (2011) A proposed unified framework for biological invasions. Trends in Ecology & Evolution 26, 333–339. 21601306 10.1016/j.tree.2011.03.023

[ref15] Bowles J, Blair D and McManus DP (1992) Genetic-variants within the genus echinococcus identified by mitochondrial-DNA sequencing. Molecular and Biochemical Parasitology 54, 165–174. 1435857 10.1016/0166-6851(92)90109-w

[ref16] Brown SP, Renaud F, Guégan J-F and Thomas F (2001) Evolution of trophic transmission in parasites: the need to reach a mating place? Journal of Evolutionary Biology 14, 815–820.

[ref17] Canadian Food Inspection (2018) Bringing animals to Canada: importing and traveling with pets. Retrieved 8 July 2021. Available at https://inspection.canada.ca/animal-health/terrestrial-animals/imports/import-policies/live-animals/pet-imports/eng/1326600389775/1326600500578.

[ref18] Catalano S, Lejeune M, Liccioli S, Verocai GG, Gesy KM, Jenkins EJ, Kutz SJ, Fuentealba C, Duignan PJ and Massolo A (2012) *Echinococcus multilocularis* in urban coyotes, Alberta, Canada. Emerging Infectious Diseases 18, 1625–1628. 23017505 10.3201/eid.1810.120119 PMC3471618

[ref19] Chubb JC, Ball MA and Parker GA (2010) Living in intermediate hosts: evolutionary adaptations in larval helminths. Trends in Parasitology 26, 93–102. 20022560 10.1016/j.pt.2009.11.008

[ref20] Cizauskas CA, Carlson CJ, Burgio KR, Clements CF, Dougherty ER, Harris NC and Phillips AJ (2017) Parasite vulnerability to climate change: an evidence-based functional trait approach. Royal Society Open Science 4, 160535. 28280551 10.1098/rsos.160535 PMC5319317

[ref21] Cleaveland S, Laurenson MK and Taylor LH (2001) Diseases of humans and their domestic mammals: pathogen characteristics, host range and the risk of emergence. Philosophical Transactions: Biological Sciences 356, 991–999. 11516377 10.1098/rstb.2001.0889 PMC1088494

[ref22] Colautti RI and MacIsaac HJ (2004) A neutral terminology to define ‘invasive’ species. Diversity and Distributions 10, 135–141.

[ref23] Cressler CE, McLeod DV, Rozins C, Van Den Hoogen J and Day T (2016) The adaptive evolution of virulence: a review of theoretical predictions and empirical tests. Parasitology 143, 915–930. 26302775 10.1017/S003118201500092X PMC4873896

[ref24] Cunningham AA, Dobson AP and Hudson PJ (2012) Disease invasion: impacts on biodiversity and human health. Philosophical Transactions of the Royal Society B: Biological Sciences 367, 2804–2806. 10.1098/rstb.2012.0331 PMC3427568 22966135

[ref25] Davidson RK, Romig T, Jenkins E, Tryland M and Robertson LJ (2012) The impact of globalisation on the distribution of *Echinococcus multilocularis*. Trends in Parasitology 28, 239–247. 22542923 10.1016/j.pt.2012.03.004

[ref26] Deplazes P and Eckert J (2001) Veterinary aspects of alveolar echinococcosis – a zoonosis of public health significance. Veterinary Parasitology 98, 65–87. 11516580 10.1016/s0304-4017(01)00424-1

[ref27] Deplazes P, Rinaldi L, Alvarez Rojas CA, Torgerson PR, Harandi MF, Romig T, Antolova D, Schurer JM, Lahmar S, Cringoli G, Magambo J, Thompson RCA and Jenkins EJ (2017) Chapter six – global distribution of alveolar and cystic echinococcosis. In Thompson RCA, Deplazes P and Lymbery AJ (eds), Echinococcus and Echinococcosis, Part A. Advances in Parasitoly, vol 95. London: Academic Press, pp. 315–493. 10.1016/bs.apar.2016.11.001 28131365

[ref28] Dlugosch KM, Anderson SR, Braasch J, Cang FA and Gillette HD (2015) The devil is in the details: genetic variation in introduced populations and its contributions to invasion. Molecular Ecology 24, 2095–2111. 25846825 10.1111/mec.13183

[ref29] Dunn AM (2009) Chapter 7 – Parasites and biological invasions. In Webster JP (ed.), Natural History of Host-Parasite Interactions. Advances in Parasitology, vol. 68. London: Academic Press, pp. 161–184.

[ref30] Dunn AM and Hatcher MJ (2015) Parasites and biological invasions: parallels, interactions, and control. Trends in Parasitology 31, 189–199. 25613560 10.1016/j.pt.2014.12.003

[ref31] Dunn AM, Torchin ME, Hatcher MJ, Kotanen PM, Blumenthal DM, Byers JE, Coon CAC, Frankel VM, Holt RD, Hufbauer RA, Kanarek AR, Schierenbeck KA, Wolfe LM and Perkins SE (2012) Indirect effects of parasites in invasions. Functional Ecology 26, 1262–1274.

[ref32] Ebert D (1995) Variation in parasite virulence is not an indicator for the evolution of benevolence. Conservation Biology 9, 1652–1653.

[ref33] Eckert J and Deplazes P (1999) Alveolar echinococcosis in humans: the current situation in Central Europe and the need for countermeasures. Parasitology Today 15, 315–319. 10407377 10.1016/s0169-4758(99)01476-3

[ref34] Eckert J, Conraths FJ and Tackmann K (2000) Echinococcosis: an emerging or re-emerging zoonosis? Thematic Issue: Emerging Parasite Zoonoses 30, 1283–1294. 10.1016/s0020-7519(00)00130-2 11113255

[ref35] Eckert J, Gemmell MA, Meslin FX and Pawlowski ZS (2001) WHO/OIE Manual on Echinococcosis in Humans and Animals: A Public Health Problem of Global Concern. Paris, France: World Organisation for Animal Health. Available from https://apps.who.int/iris/handle/10665/42427.

[ref36] Essl F, Lenzner B, Bacher S, Bailey S, Capinha C, Daehler C, Dullinger S, Genovesi P, Hui C, Hulme PE, Jeschke JM, Katsanevakis S, Kühn I, Leung B, Liebhold A, Liu C, MacIsaac HJ, Meyerson LA, Nuñez MA, Pauchard A, Pyšek P, Rabitsch W, Richardson DM, Roy HE, Ruiz GM, Russell JC, Sanders NJ, Sax DF, Scalera R, Seebens H, Springborn M, Turbelin A, van Kleunen M, von Holle B, Winter M, Zenni RD, Mattsson BJ and Roura-Pascual N (2020) Drivers of future alien species impacts: an expert-based assessment. Global Change Biology 26, 4880–4893. 32663906 10.1111/gcb.15199 PMC7496498

[ref37] Ewald PW (1995) The evolution of virulence: a unifying link between parasitology and ecology. The Journal of Parasitology 81, 659–669. 7472852

[ref38] Feis ME, Goedknegt MA, Thieltges DW, Buschbaum C and Wegner KM (2016) Biological invasions and host–parasite coevolution: different co-evolutionary trajectories along separate parasite invasion fronts. Zoology 119, 366–374. 27373339 10.1016/j.zool.2016.05.012

[ref39] Frankham R (2004) Resolving the genetic paradox in invasive species. Heredity 94, 385. 10.1038/sj.hdy.6800634 15602569

[ref40] Fuglei E and Tarroux A (2019) Arctic fox dispersal from Svalbard to Canada: one female's long run across sea ice. Polar Research 38, 1–7.

[ref41] Georgiev B, Sánchez M, Vasileva G, Nikolov P and Green A (2007) Cestode parasitism in invasive and native brine shrimps (*Artemia* spp.) as a possible factor promoting the rapid invasion of *A. franciscana* in the Mediterranean region. Parasitology Research 101, 1647–1655. 17712569 10.1007/s00436-007-0708-3

[ref42] Gesy KM and Jenkins EJ (2015) Introduced and native haplotypes of *Echinococcus multilocularis* in wildlife in Saskatchewan, Canada. Journal of Wildlife Diseases 51, 743–748. 26020284 10.7589/2014-08-214

[ref43] Gesy K, Hill JE, Schwantje S, Liccioli S and Jenkins EJ (2013) Establishment of a European-type strain of *Echinococcus multilocularis* in Canadian wildlife. Parasitology 140, 1133–1137. 23714582 10.1017/S0031182013000607

[ref44] Gesy KM, Schurer JM, Massolo A, Liccioli S, Elkin BT, Alisauskas R and Jenkins EJ (2014) Unexpected diversity of the cestode *Echinococcus multilocularis* in wildlife in Canada. International Journal for Parasitology: Parasites and Wildlife 3, 81–87. 25161905 10.1016/j.ijppaw.2014.03.002 PMC4142260

[ref45] Giraudoux P, Craig PS, Delattre P, Bao G, Bartholomot B, Harraga S, Quere JP, Raoul F, Wang Y, Shi D and Vuitton DA (2003) Interactions between landscape changes and host communities can regulate *Echinococcus multilocularis* transmission. Parasitology 127(S1), S121–S131. 15027609

[ref46] Godfrey SS (2013) Networks and the ecology of parasite transmission: a framework for wildlife parasitology. International Journal for Parasitology: Parasites and Wildlife 2, 235–245. 24533342 10.1016/j.ijppaw.2013.09.001 PMC3862525

[ref47] Gompper ME (2002) Top carnivores in the suburbs? Ecological and conservation issues raised by colonization of north-eastern North America by coyotes: the expansion of the coyote's geographical range may broadly influence community structure, and rising coyote densities in the suburbs may alter how the general public views wildlife. Bioscience 52, 185–190.

[ref48] Gosselink TE, Van Deelen TR, Warner RE and Joselyn MG (2003) Temporal habitat partitioning and spatial use of coyotes and red foxes in East-central Illinois. The Journal of Wildlife Management 67, 90–103.

[ref49] Hatcher MJ, Dick JTA and Dunn AM (2006) How parasites affect interactions between competitors and predators. Ecology Letters 9, 1253–1271. 17040328 10.1111/j.1461-0248.2006.00964.x

[ref50] Hatcher MJ, Dick JT and Dunn AM (2012 *a*) Diverse effects of parasites in ecosystems: linking interdependent processes. Frontiers in Ecology and the Environment 10, 186–194.

[ref51] Hatcher MJ, Dick JTA and Dunn AM (2012 *b*) Disease emergence and invasions. Functional Ecology 26, 1275–1287. 32313353 10.1111/j.1365-2435.2012.02031.x PMC7163950

[ref52] Henttonen H, Fuglei E, Gower CN, Haukisalmi V, Ims RA, Niemimaa J and Yoccoz NG (2001) *Echinococcus multilocularis* on Svalbard: introduction of an intermediate host has enabled the local lifecycle. Parasitology 123, 547–552. 11814041 10.1017/s0031182001008800

[ref53] Hoberg EP (2010) Invasive processes, mosaics and the structure of helminth parasite faunas. Revue Scientifique et Technique 29, 255–272. 20919581

[ref54] Hoberg EP and Brooks DR (2015) Evolution in action: climate change, biodiversity dynamics and emerging infectious disease. Philosophical Transactions of the Royal Society of London B Biological Sciences 370, 1–7. 10.1098/rstb.2013.0553 PMC4342959 25688014

[ref55] Houston S, Belga S, Buttenschoen K, Cooper R, Girgis S, Gottstein B, Low G, Massolo A, MacDonald C, Müller N, Preiksaitis J, Sarlieve P, Vaughan S and Kowalewska-Grochowska K (2021) Epidemiological and clinical characteristics of alveolar echinococcosis: an emerging infectious disease in Alberta, Canada. The American Journal of Tropical Medicine and Hygiene 104, 1863–1869. 10.4269/ajtmh.20-1577 PMC8103444 33755579

[ref56] Hudson P and Greenman J (1998) Competition mediated by parasites: biological and theoretical progress. Trends in Ecology & Evolution 13, 387–390. 21238357 10.1016/s0169-5347(98)01475-x

[ref57] Janzen DH (1985) On ecological fitting. Oikos 45, 308–310.

[ref58] Jenkins EJ, Peregrine AS, Hill JE, Somers C, Gesy K, Barnes B, Gottstein B and Polley L (2012) Detection of European strain of *Echinococcus multilocularis* in North America. Emerging Infectious Diseases 18, 1010–1012. 22608114 10.3201/eid1806.111420 PMC3358155

[ref59] Jephcott TG, Sime-Ngando T, Gleason FH and Macarthur DJ (2016) Host–parasite interactions in food webs: diversity, stability, and coevolution. Food Webs 6, 1–8.

[ref60] Johnson PTJ and Thieltges DW (2010) Diversity, decoys and the dilution effect: how ecological communities affect disease risk. The Journal of Experimental Biology 213, 961–970. 20190121 10.1242/jeb.037721

[ref61] Jones KE, Patel NG, Levy MA, Storeygard A, Balk D, Gittleman JL and Daszak P (2008) Global trends in emerging infectious diseases. Nature 451, 990–993. 18288193 10.1038/nature06536 PMC5960580

[ref62] Julien DA, Sargeant JM, Filejski C and Harper SL (2021) Who let the dogs in? An epidemiological study quantifying domestically sourced and imported dogs in Southern Ontario, Canada. Zoonoses and Public Health 1–13. doi: 10.1111/zph.12847 33987921

[ref63] Kaltz O and Shykoff JA (1998) Local adaptation in host–parasite systems. Heredity 81, 361.

[ref64] Kamler JF and Ballard WB (2002) A review of native and nonnative red foxes in North America. Wildlife Society Bulletin 30, 370–379.

[ref65] Kapel CMO, Torgerson PR, Thompson RCA and Deplazes P (2006) Reproductive potential of *Echinococcus multilocularis* in experimentally infected foxes, dogs, raccoon dogs and cats. International Journal for Parasitology 36, 79–86. 16199043 10.1016/j.ijpara.2005.08.012

[ref66] Keesing F, Belden LK, Daszak P, Dobson A, Harvell CD, Holt RD, Hudson P, Jolles A, Jones KE, Mitchell CE, Myers SS, Bogich T and Ostfeld RS (2010) Impacts of biodiversity on the emergence and transmission of infectious diseases. Nature 468, 647. 21124449 10.1038/nature09575 PMC7094913

[ref67] Kelly DW, Paterson RA, Townsend CR, Poulin R and Tompkins DM (2009) Parasite spillback: a neglected concept in invasion ecology? Ecology 90, 2047–2056. 19739367 10.1890/08-1085.1

[ref68] Kinkar L, Laurimäe T, Acosta-Jamett G, Andresiuk V, Balkaya I, Casulli A, Gasser RB, van der Giessen J, González LM, Haag KL, Zait H, Irshadullah M, Jabbar A, Jenkins DJ, Kia EB, Manfredi MT, Mirhendi H, M'Rad S, Rostami-Nejad M, Oudni-M'rad M, Pierangeli NB, Ponce-Gordo F, Rehbein S, Sharbatkhori M, Simsek S, Soriano SV, Sprong H, Šnábel V, Umhang G, Varcasia A and Saarma U (2018 *a*) Global phylogeography and genetic diversity of the zoonotic tapeworm *Echinococcus granulosus* sensu stricto genotype G1. International Journal for Parasitology 48, 729–742. 29782829 10.1016/j.ijpara.2018.03.006

[ref69] Kinkar L, Laurimäe T, Balkaya I, Casulli A, Zait H, Irshadullah M, Sharbatkhori M, Mirhendi H, Rostami-Nejad M, Ponce-Gordo F, Rehbein S, Kia EB, Simsek S, Šnábel V, Umhang G, Varcasia A and Saarma U (2018 *b*) Genetic diversity and phylogeography of the elusive, but epidemiologically important *Echinococcus granulosus* sensu stricto genotype G3. Parasitology 145, 1613–1622. 29661261 10.1017/S0031182018000549

[ref70] Kirk RS (2003) The impact of *Anguillicola crassus* on European eels. Fisheries Management and Ecology 10, 385–394.

[ref71] Klein C and Massolo A (2015) Demonstration that a case of human alveolar echinococcosis in Minnesota in 1977 was caused by the N2 strain. American Journal of Tropical Medicine and Hygiene 92, 477–478. 10.4269/ajtmh.14-0484 PMC4350532 25404078

[ref72] Knapp J, Guislain MH, Bart JM, Raoul F, Gottstein B, Giraudoux P and Piarroux R (2008) Genetic diversity of *Echinococcus multilocularis* on a local scale. Infection, Genetics and Evolution 8, 367–373. 10.1016/j.meegid.2008.02.010 18406214

[ref73] Knapp J, Bart J-M, Giraudoux P, Glowatzki M-L, Breyer I, Raoul F, Deplazes P, Duscher G, Martinek K, Dubinsky P, Guislain M-H, Cliquet F, Romig T, Malczewski A, Gottstein B and Piarroux R (2009) Genetic diversity of the cestode *Echinococcus multilocularis* in red foxes at a continental scale in Europe. PLoS Neglected Tropical Diseases 3, e452. 19513103 10.1371/journal.pntd.0000452 PMC2685985

[ref74] Knapp J, Bart JM, Maillard S, Gottstein B and Piarroux R (2010) The genomic *Echinococcus microsatellite* EmsB sequences: from a molecular marker to the epidemiological tool. Parasitology 137, 439–449. 20025824 10.1017/S0031182009991612

[ref75] Kolar CS and Lodge DM (2001) Progress in invasion biology: predicting invaders. Trends in Ecology & Evolution 16, 199–204. 11245943 10.1016/s0169-5347(01)02101-2

[ref76] Kotwa JD, Isaksson M, Jardine CM, Campbell GD, Berke O, Pearl DL, Mercer NJ, Osterman-Lind E and Peregrine AS (2019) *Echinococcus multilocularis* infection, Southern Ontario, Canada. Emerging Infectious Diseases 25, 265–272. 30666935 10.3201/eid2502.180299 PMC6346450

[ref77] Krakau M, Thieltges DW and Reise K (2006) Native parasites adopt introduced bivalves of the North Sea. Biological Invasions 8, 919.

[ref78] Leggett HC, Brown SP and Reece SE (2014) War and peace: social interactions in infections. Philosophical Transactions of the Royal Society B: Biological Sciences 369, 20130365. 10.1098/rstb.2013.0365 PMC3982666 24686936

[ref79] Lello J, Boag B, Fenton A, Stevenson IR and Hudson PJ (2004) Competition and mutualism among the gut helminths of a mammalian host. Nature 428, 840. 15103373 10.1038/nature02490

[ref80] Levi T, Keesing F, Holt RD, Barfield M and Ostfeld RS (2016) Quantifying dilution and amplification in a community of hosts for tick-borne pathogens. Ecological Applications 26, 484–498. 27209790 10.1890/15-0122

[ref81] Liccioli S, Catalano S, Kutz SJ, Lejeune M, Verocai GG, Duignan PJ, Fuentealba C, Hart M, Ruckstuhl KE and Massolo A (2012) Gastrointestinal parasites of coyotes (*Canis latrans*) in the metropolitan area of Calgary, Alberta, Canada. Canadian Journal of Zoology-Revue Canadienne De Zoologie 90, 1023–1030.

[ref82] Liccioli S, Duignan PJ, Lejeune M, Deunk J, Majid S and Massolo A (2013) A new intermediate host for *Echinococcus multilocularis*: the southern red-backed vole (*Myodes gapperi*) in urban landscape in Calgary, Canada. Parasitology International 62, 355–357. 23608104 10.1016/j.parint.2013.03.007

[ref83] Liccioli S, Kutz SJ, Ruckstuhl KE and Massolo A (2014) Spatial heterogeneity and temporal variations in *Echinococcus multilocularis* infections in wild hosts in a North American urban setting. International Journal for Parasitology 44, 457–465. 24747533 10.1016/j.ijpara.2014.03.007

[ref84] Liccioli S, Bialowas C, Ruckstuhl KE and Massolo A (2015 *a*) Feeding ecology informs parasite epidemiology: prey selection modulates encounter rate with *Echinococcus multilocularis* in urban coyotes. PLoS One 10, e0121646. 25768437 10.1371/journal.pone.0121646 PMC4359113

[ref85] Liccioli S, Giraudoux P, Deplazes P and Massolo A (2015 *b*) Wilderness in the ‘city’ revisited: different urbes shape transmission of *Echinococcus multilocularis* by altering predator and prey communities. Trends in Parasitology 31, 297–305. 25985897 10.1016/j.pt.2015.04.007

[ref86] Lockwood JL, Cassey P and Blackburn T (2005) The role of propagule pressure in explaining species invasions. Trends in Ecology & Evolution 20, 223–228. 16701373 10.1016/j.tree.2005.02.004

[ref87] Lockwood JL, Hoopes MF and Marchetti MP (2013) Invasion Ecology, 2nd Edn. Chichester, UK: Wiley-Blackwell.

[ref88] Lymbery AJ (2017) Phylogenetic pattern, evolutionary processes and species delimitation in the genus *Echinococcus*. In Thompson RCA, Deplazes P and Lymbery AJ (eds), Echinococcus and Echinococcosis, Part A. Advances in Parasitology, vol 95. London: Academic Press, pp. 111–145. 10.1016/bs.apar.2016.07.002 28131362

[ref89] Lymbery AJ, Morine M, Kanani HG, Beatty SJ and Morgan DL (2014) Co-invaders: the effects of alien parasites on native hosts. International Journal of Parasitol: Parasites and Wildlife 3, 171–177. 10.1016/j.ijppaw.2014.04.002 PMC4145144 25180161

[ref90] Malcicka M, Agosta SJ and Harvey JA (2015) Multi level ecological fitting: indirect life cycles are not a barrier to host switching and invasion. Global Change Biology 21, 3210–3218. 25778909 10.1111/gcb.12928

[ref91] Massolo A, Liccioli S, Budke C and Klein C (2014) *Echinococcus multilocularis* in North America: the great unknown. Parasite 21, 73. 25531581 10.1051/parasite/2014069 PMC4273702

[ref92] Massolo A, Valli D, Wassermann M, Cavallero S, D'Amelio S, Meriggi A, Torretta E, Serafini M, Casulli A, Zambon L, Boni CB, Ori M, Romig T and Macchioni F (2018) Unexpected *Echinococcus multilocularis* infections in shepherd dogs and wolves in south-western Italian Alps: a new endemic area? International Journal for Parasitology: Parasites and Wildlife 7, 309–316. 30175043 10.1016/j.ijppaw.2018.08.001 PMC6115541

[ref93] Massolo A, Klein C, Kowalewska-Grochowska K, Belga S, MacDonald C, Vaughan S, Girgis S, Giunchi D, Bramer SA, Santa MA, Grant DM, Mori K, Duignan P, Slater O, Gottstein B, Müller N and Houston S (2019) European *Echinococcus multilocularis* identified in patients in Canada. New Englamd Journal of Medicine 381, 384–385. 10.1056/NEJMc1814975 31340100

[ref94] McCreesh N, Nikulin G and Booth M (2015) Predicting the effects of climate change on *Schistosoma mansoni* transmission in Eastern Africa. Parasites & Vectors 8, 4. 25558917 10.1186/s13071-014-0617-0 PMC4297451

[ref95] Mideo N (2009) Parasite adaptations to within-host competition. Trends in Parasitology 25, 261–268. 19409846 10.1016/j.pt.2009.03.001

[ref96] Molnár PK, Dobson AP and Kutz SJ (2013) Gimme shelter – the relative sensitivity of parasitic nematodes with direct and indirect life cycles to climate change. Global Change Biology 19, 3291–3305. 23801641 10.1111/gcb.12303

[ref97] Moore J (2002) Parasites and the Behavior of Animals. New York: Oxford University Press.

[ref98] Nackley LL, West AG, Skowno AL and Bond WJ (2017) The nebulous ecology of native invasions. Trends in Ecology & Evolution 32, 814–824. 28890126 10.1016/j.tree.2017.08.003

[ref99] Nakao M, Sako Y and Ito A (2003) Isolation of polymorphic microsatellite loci from the tapeworm *Echinococcus multilocularis*. Infection, Genetics and Evolution 3, 159–163. 10.1016/s1567-1348(03)00070-4 14522179

[ref100] Nakao M, Xiao N, Okamoto M, Yanagida T, Sako Y and Ito A (2009) Geographic pattern of genetic variation in the fox tapeworm *Echinococcus multilocularis*. Parasitology International 58, 384–389. 19651237 10.1016/j.parint.2009.07.010

[ref101] Obayashi M, Rausch RL and Fay FH (1971) On the ecology and distribution of *Echinococcus* spp. (Cestoda: Taeniidae), and characteristics of their development in the intermediate host. II. Comparative studies on the development of larval *E. multilocularis* leuckart, 1863, in the intermediate host. The Japanese Journal of Veterinary Research 19(Suppl 3), 1–53. 5315064

[ref102] Ogden NH, Wilson JRU, Richardson DM, Hui C, Davies SJ, Kumschick S, Le Roux JJ, Measey J, Saul W-C and Pulliam JRC (2019) Emerging infectious diseases and biological invasions: a call for a one health collaboration in science and management. Royal Society Open Science 6, 181577–181577. 31032015 10.1098/rsos.181577 PMC6458372

[ref103] Oscos-Snowball A, Tan E, Peregrine AS, Foster R, Bronsoiler J, Gottstein B, Jenkins E, Gesy K and Bienzle D (2015) What is your diagnosis? Fluid aspirated from an abdominal mass in a dog. Veterinary Clinical Pathology 44, 167–168. 25352067 10.1111/vcp.12210

[ref104] Ostfeld RS and Keesing F (2012) Effects of host diversity on infectious disease. Annual Review of Ecology, Evolution, and Systematics 43, 157–182.

[ref105] Parker GA, Chubb JC, Ball MA and Roberts GN (2003) Evolution of complex life cycles in helminth parasites. Nature 425, 480. 14523438 10.1038/nature02012

[ref106] Parker IM, Saunders M, Bontrager M, Weitz AP, Hendricks R, Magarey R, Suiter K and Gilbert GS (2015) Phylogenetic structure and host abundance drive disease pressure in communities. Nature 520, 542. 25903634 10.1038/nature14372

[ref107] Peregrine AS (2015) Alveolar echinococcosis in dogs: an emerging issue? Veterinary Record 177, 567–567. 10.1136/vr.h6551 26637618

[ref108] Poulin R and Maure F (2015) Host manipulation by parasites: a look back before moving forward. Trends in Parasitology 31, 563–570. 26440784 10.1016/j.pt.2015.07.002

[ref109] Prenter J, Macneil C, Dick JT and Dunn AM (2004) Roles of parasites in animal invasions. Trends in Ecology and Evolution 19, 385–390. 16701290 10.1016/j.tree.2004.05.002

[ref110] Rausch RL and Richards SH (1971) Observations on parasite–host relationships of *Echinococcus multilocularis* Leuckart, 1863, in North Dakota. Canadian Journal of Zoology 49, 1317–1330. 5119812 10.1139/z71-198

[ref111] Read AF and Taylor LH (2001) The ecology of genetically diverse infections. Science (New York, N.Y.) 292, 1099–1102. 10.1126/science.1059410 11352063

[ref112] Ricciardi A and Cohen J (2007) The invasiveness of an introduced species does not predict its impact. Biological Invasions 9, 309–315.

[ref113] Rius M and Darling JA (2014) How important is intraspecific genetic admixture to the success of colonising populations? Trends in Ecology & Evolution 29, 233–242. 24636862 10.1016/j.tree.2014.02.003

[ref114] Rohr JR, Civitello DJ, Halliday FW, Hudson PJ, Lafferty KD, Wood CL and Mordecai EA (2020) Towards common ground in the biodiversity–disease debate. Nature Ecology & Evolution 4, 24–33. 31819238 10.1038/s41559-019-1060-6 PMC7224049

[ref115] Roman J and Darling JA (2007) Paradox lost: genetic diversity and the success of aquatic invasions. Trends in Ecology and Evolution 22, 454–464. 17673331 10.1016/j.tree.2007.07.002

[ref116] Romig T, Deplazes P, Jenkins D, Giraudoux P, Massolo A, Craig PS, Wassermann M, Takahashi K and de la Rue M (2017) Ecology and life cycle patterns of *Echinococcus* species. In Thompson RCA, Deplazes P, Lymbery AJ (eds), Echinococcus and Echinococcosis, Part A. Advances in Parasitoly, vol. 95. London: Academic Press, ch. 5, pp. 213–314. 10.1016/bs.apar.2016.11.002 28131364

[ref117] Santa MA, Pastran SA, Klein C, Duignan P, Ruckstuhl K, Romig T and Massolo A (2018) Detecting co-infections of *Echinococcus multilocularis* and *Echinococcus canadensis* in coyotes and red foxes in Alberta, Canada using real-time PCR. International Journal for Parasitology: Parasites and Wildlife 7, 111–115. 29988802 10.1016/j.ijppaw.2018.03.001 PMC6031960

[ref118] Santa MA, Rezansoff AM, Chen R, Gilleard JS, Musiani M, Ruckstuhl KE and Massolo A (2021) Deep amplicon sequencing highlights low intra-host genetic variability of *Echinococcus multilocularis* and high prevalence of the European-type haplotypes in coyotes and red foxes in Alberta, Canada. PLOS Neglected Tropical Diseases 15, e0009428. 34038403 10.1371/journal.pntd.0009428 PMC8153462

[ref119] Schrieber K and Lachmuth S (2017) The genetic paradox of invasions revisited: the potential role of inbreeding × environment interactions in invasion success. Biological Reviews Cambridge Philosophical Society 92, 939–952. 10.1111/brv.12263 27009691

[ref120] Schurer JM, Gesy KM, Elkin BT and Jenkins EJ (2013) *Echinococcus multilocularis* and *Echinococcus canadensis* in wolves from western Canada. Parasitology 141, 159–163. 24135428 10.1017/S0031182013001716

[ref121] Schurer JM, Pawlik M, Huber A, Elkin B, Cluff HD, Pongracz JD, Gesy K, Wagner B, Dixon B, Merks H, Bal MS and Jenkins EJ (2016) Intestinal parasites of gray wolves (*Canis lupus*) in northern and western Canada. Canadian Journal of Zoology 94, 643–650.

[ref122] Schurer JM, Tsybina P, Gesy KM, Kolapo TU, Skinner S, Hill JE and Jenkins EJ (2020) Molecular evidence for local acquisition of human alveolar echinococcosis in Saskatchewan, Canada. The Journal of Infectious Diseases 223, 1015–1018. 10.1093/infdis/jiaa473 32766836

[ref123] Seppälä O and Jokela J (2016) Do coinfections maintain genetic variation in parasites? Trends in Parasitology 32, 930–938. 27614425 10.1016/j.pt.2016.08.010

[ref124] Shi J, Joshi J, Tielbörger K, Verhoeven KJF and Macel M (2018) Costs and benefits of admixture between foreign genotypes and local populations in the field. Ecology and Evolution 8, 3675–3684. 29686848 10.1002/ece3.3946 PMC5901173

[ref125] Skelding A, Brooks A, Stalker M, Mercer N, de Villa E, Gottstein B and Peregrine AS (2014) Hepatic alveolar hydatid disease (*Echinococcus multilocularis*) in a boxer dog from southern Ontario. The Canadian Veterinary Journal 55, 551. 24891637 PMC4022022

[ref126] Sokurenko EV, Gomulkiewicz R and Dykhuizen DE (2006) Source–sink dynamics of virulence evolution. Nature Reviews Microbiology 4, 548–555. 16778839 10.1038/nrmicro1446

[ref127] Stensgaard A-S, Vounatsou P, Sengupta ME and Utzinger J (2019) Schistosomes, snails and climate change: current trends and future expectations. Acta Tropica 190, 257–268. 30261186 10.1016/j.actatropica.2018.09.013

[ref128] Strauss A, White A and Boots M (2012) Invading with biological weapons: the importance of disease-mediated invasions. Functional Ecology 26, 1249–1261.

[ref129] Thompson RCA (2017) Biology and systematics of echinococcus. Advances in Parasitology 95, 65–109. 28131366 10.1016/bs.apar.2016.07.001

[ref130] Toews E, Musiani M, Checkley S, Visscher D and Massolo A (2021) A global assessment of Echinococcus multilocularis infections in domestic dogs: proposing a framework to overcome past methodological heterogeneity. International Journal for Parasitology 51, 379–392. 33482171 10.1016/j.ijpara.2020.10.008

[ref131] Tompkins DM, Sainsbury AW, Nettleton P, Buxton D and Gurnell J (2002) *Parapoxvirus* causes a deleterious disease in red squirrels associated with UK population declines. Proceedings: Biological Sciences 269, 529–533. 11886647 10.1098/rspb.2001.1897 PMC1690913

[ref132] Tompkins DM, Carver S, Jones ME, Krkošek M and Skerratt LF (2015) Emerging infectious diseases of wildlife: a critical perspective. Trends in Parasitology 31, 149–159. 25709109 10.1016/j.pt.2015.01.007

[ref133] Torchin ME, Lafferty KD, Dobson AP, McKenzie VJ and Kuris AM (2003) Introduced species and their missing parasites. Nature 421, 628–630. 12571595 10.1038/nature01346

[ref134] Torgerson PR and Craig PS (2009) Risk assessment of importation of dogs infected with *Echinococcus multilocularis* into the UK. Veterinary Record 165, 366–368. 10.1136/vr.165.13.366 19783849

[ref135] Umhang G, Karamon J, Hormaz V, Knapp J, Cencek T and Boué F (2017) A step forward in the understanding of the presence and expansion of *Echinococcus multilocularis* in Eastern Europe using microsatellite EmsB genotyping in Poland. Infection, Genetics and Evolution 54, 176–182. 10.1016/j.meegid.2017.07.004 28688974

[ref136] van Paridon BJ, Colwell DD, Goater CP and Gilleard JS (2017) Population genetic analysis informs the invasion history of the emerging trematode *Dicrocoelium dendriticum* into Canada. International Journal for Parasitology 47, 845–856. 28668324 10.1016/j.ijpara.2017.04.006

[ref137] Verhoeven KJ, Macel M, Wolfe LM and Biere A (2011) Population admixture, biological invasions and the balance between local adaptation and inbreeding depression. Proceedings of the Royal Society Biological Sciences 278, 2–8. 20685700 10.1098/rspb.2010.1272 PMC2992731

[ref138] Vervaeke M, Davis S, Leirs H and Verhagen R (2006) Implications of increased susceptibility to predation for managing the sylvatic cycle of *Echinococcus multilocularis*. Parasitology 132, 893–901. 16454866 10.1017/S0031182006009838

[ref139] Villeneuve A, Polley L, Jenkins E, Schurer J, Gilleard J, Kutz S, Conboy G, Benoit D, Seewald W and Gagné F (2015) Parasite prevalence in fecal samples from shelter dogs and cats across the Canadian provinces. Parasites & Vectors 8, 1–10. 26013283 10.1186/s13071-015-0870-x PMC4451884

[ref140] Walther G-R, Roques A, Hulme PE, Sykes MT, Pyšek P, Kühn I, Zobel M, Bacher S, Botta-Dukát Z, Bugmann H, Czúcz B, Dauber J, Hickler T, Jarošík V, Kenis M, Klotz S, Minchin D, Moora M, Nentwig W, Ott J, Panov VE, Reineking B, Robinet C, Semenchenko V, Solarz W, Thuiller W, Vilà M, Vohland K and Settele J (2009) Alien species in a warmer world: risks and opportunities. Trends in Ecology & Evolution 24, 686–693. 19712994 10.1016/j.tree.2009.06.008

[ref141] Walton LR, Cluff HD, Paquet PC and Ramsay MA (2001) Movement patterns of barren-ground wolves in the central Canadian Arctic. Journal of Mammalogy 82, 867–876.

[ref142] Young HS, Parker IM, Gilbert GS, Sofia Guerra A and Nunn CL (2017) Introduced species, disease ecology, and biodiversity–disease relationships. Trends in Ecology & Evolution 32, 41–54. 28029377 10.1016/j.tree.2016.09.008

[ref143] Zhou X-N, Yang G-J, Yang K, Wang X-H, Hong Q-B, Sun L-P, Malone JB, Kristensen TK, Bergquist NR and Utzinger J (2008) Potential impact of climate change on schistosomiasis transmission in China. The American Journal of Tropical Medicine and Hygiene 78, 188–194. 18256410

[ref144] Ziadinov I, Mathis A, Trachsel D, Rysmukhambetova A, Abdyjaparov TA, Kuttubaev OT, Deplazes P and Torgerson PR (2008) Canine echinococcosis in Kyrgyzstan: using prevalence data adjusted for measurement error to develop transmission dynamics models. International Journal for Parasitology 38, 1179–1190. 18371969 10.1016/j.ijpara.2008.01.009 PMC2527539

