# Supplementary material for: A review on invasions by parasites with complex life cycles: the European strain of Echinococcus multilocularis in North America as a model
Source: Parasitology. 2021 Aug 18;148(13):1532–44. doi: 10.1017/S0031182021001426 (PMC8564803; doi:10.1017/S0031182021001426)
Supplement: Supplementary file 1 [file S0031182021001426sup001.docx]

**S1 Glossary:**

**Complex life cycle parasite:** the parasite that relies on more than one host species, usually in a particular sequence, to complete its life cycle.

**Drift loads:** stochastic increases in the frequency of deleterious alleles (i.e. due to small population size in bottlenecked invasive populations).

**Emerging infectious diseases:** a disease that has appeared in a population for the first time, or that may have existed previously but is rapidly increasing in incidence or geographical range.

**Endemism:** in epidemiology, it is the continuous presence of a disease or infectious agent in a given geographic area, or population group.

**Genetic admixture:** reproduction between individuals from different ancestral populations resulting in introducing new genetic lineages into a population.

**Genetic depletion**: loss of particular alleles or genes, and consequent reduction of genetic diversity.

**Haplotype:** specific combination of alleles for different polymorphisms that occur on the same chromosome and inherited together from a single parent.

**Host specificity:** range and diversity of host species that a parasite is capable of infecting.

**Host-switching:** when parasites jump to and become established in a new host species creating new host-parasite associations.

**Inbreeding depression:** decrease in fitness with increased genome-wide homozygosity that occurs in the offspring of related parents.

**Infectivity**: likelihood that an infectious agent will infect a host, given that the host is exposed to the agent.

**Intraguild predation:** when a dominant predator selectively kills subordinate rivals to gain increased access to resources.

**Outbreak:** when a disease occurs in greater numbers than expected in a community or region or during a season.

**Parasite:** organisms that live in or on, and at the expense of other organisms (hosts), resulting in harm to the host.

**Phenotypic plasticity:** the ability of a genotype to express different phenotypes in different environments.

**Propagule:** any structure capable of being propagated or acting as an agent of reproduction.

**Reverse phototaxis:** negative displacement along a light gradient or vector.

**Vector:** any organism (vertebrate or invertebrate) that functions as a carrier of an infectious agent between organisms of a different species.

**Virulence:** the ability of an infectious agent to cause disease in the host resulting in decreased host fitness. Parasite-mediated morbidity and mortality.
